# Region-Specific Growth Effects in the Developing Rat Prostate Following Fetal Exposure to Estrogenic Ultraviolet Filters

**DOI:** 10.1289/ehp.10983

**Published:** 2008-03-25

**Authors:** Luke Hofkamp, Sarahann Bradley, Jesus Tresguerres, Walter Lichtensteiger, Margret Schlumpf, Barry Timms

**Affiliations:** 1 Division of Basic Biomedical Sciences, Sanford School of Medicine, University of South Dakota, Vermillion, South Dakota, USA; 2 Department of Physiology, School of Medicine, Complutense University, Madrid, Spain; 3 GREEN Tox and Institute of Anatomy, University of Zürich, Zürich, Switzerland

**Keywords:** endocrine disruptors, 4-methylbenzylidene camphor (4-MBC), prostate development, UV filters

## Abstract

**Background and objectives:**

Exposure to environmental endocrine disruptors is a potential risk factor for humans. Many of these chemicals have been shown to exhibit disruption of normal cellular and developmental processes in animal models. Ultraviolet (UV) filters used as sunscreens in cosmetics have previously been shown to exhibit estrogenic activity in *in vitro* and *in vivo* assays. We examined the effects of two UV filters, 4-methylbenzylidene camphor (4-MBC) and 3-benzylidene camphor (3-BC), in the developing prostate of the fetal rat.

**Methods:**

Pregnant Long Evans rats were fed diets containing doses of 4-MBC and 3-BC that resulted in average daily intakes of these chemicals corresponding to the lowest observed adverse effects level (LOAEL) and the no observed adverse effects level (NOAEL) doses in prior developmental toxicity studies. Using digital photographs of serial sections from postnatal day 1 animals, we identified, contoured, and aligned the epithelial ducts from specific regions of the developing prostate, plus the accessory sex glands and calculated the total volume for each region from three-dimensional, surface-rendered models.

**Results:**

Fetal exposure to 4-MBC (7.0 mg/kg body weight/day) resulted in a significant increase (*p* < 0.05) in tissue volume in the prostate and accessory sex glands. Treated males exhibited a 62% increase in the number of ducts in the caudal dorsal prostate. Increased distal branching morphogenesis appears to be a consequence of exposure in the ventral region, resulting in a 106% increase in ductal volume.

**Conclusions:**

4-MBC exposure during development of the male reproductive accessory sex glands exhibited classical growth effects associated with estrogenic endocrine disruptors. The different regional responses suggest that the two developmental processes of ductal outgrowth and branching morphogenesis are affected independently by exposure to the environmental chemicals.

Exposure to environmental endocrine disruptors is a potential risk factor for humans. With the increasing use of ultraviolet (UV) filters in sunscreens and cosmetics and as additives in plastics and household products, their possible environmental impact deserves consideration. UV filters may be directly introduced into surface waters during swimming or they may enter wastewater from households or industry. There is good evidence that pharmaceuticals and ingredients of personal care products can spread into the biosphere and reach the food chain: UV filters are present in water downstream of sewage treatment plants (Kupper et al. 2006; Plagellat et al. 2006) in surface waters (Buser et al. 2005, 2006), and they bioaccumulate in fish (Balmer et al. 2005, Buser et al. 2006; Nagtegaal et al. 1997). In a recent monitoring study, UV filters were found in 75% of human milk samples (Schlumpf et al. 2008), indicating potential exposure of newborns.

Certain UV filters have been shown to exhibit estrogenic activity in both *in vitro* and *in vivo* assays (Schlumpf et al. 2001, 2004a), and they have also shown developmental toxicity (Durrer et al. 2005, 2007; Maerkel et al. 2007). *In vitro* studies showed that 4-methyl-benzylidene camphor (4-MBC) and 3-benzylidene camphor (3-BC), are estrogen receptor (ER)-β ligands (Schlumpf et al. 2004a), but they are also active in ER-α–typical *in vivo* tests such as the uterotrophic assay (Schlumpf et al. 2001, 2004a).

Many endocrine-active xenobiotics affect normal cellular and developmental processes in animal models, with the prostate being one sensitive target (Timms et al. 2005; vom Saal et al. 1997). These effects can be seen in both the juvenile and adult stages of growth in the prostate. Developmental exposure to 4-MBC and 3-BC affects prostate weight and estrogen target gene expression, in addition to effects on other targets such as brain, uterus, and thyroid (Durrer et al. 2005, 2007; Maerkel et al. 2007; Schlumpf et al. 2004b).

Although fetal prostate development is primarily regulated by androgens (Siiteri and Wilson 1974), addition of exogenous estrogens during critical periods of growth has the potential to alter the development of the prostate (Richter et al. 2007; Timms et al. 2005; vom Saal et al. 1997). Growth responses to estrogen and estrogen mimics are variable and depend on dose and timing of treatment. Low-level exposure to estrogenic compounds administered during fetal development results in a region-specific proliferative growth response (Timms et al. 2005; vom Saal et al. 1997). Conversely, postnatal treatment with higher doses of estrogen has an inhibitory effect on growth (Huang et al. 2005; Prins and Korach 2007; Timms et al. 2005).

In the present study we investigated the effects of 4-MBC and 3-BC on the developing prostate in the fetal rat. This experiment was designed, as previously described (Durrer et al. 2005, 2007; Maerkel et al. 2007), to examine the effects caused by systemic, low-dose exposure throughout *in utero* development. We administered 4-MBC and 3-BC at two dose levels, corresponding to previously published classical lowest observed adverse effect level (LOAEL) and classical no observed adverse effect level (NOAEL)/molecular LOAEL, respectively (Durrer et al. 2005, 2007; Maerkel et al. 2007; Schlumpf et al. 2008). Our data support the hypothesis that exposure to low doses of environmentally relevant endocrine disruptors during early prostate development alters normal growth patterns and extends the range of active chemicals to UV filters used in cosmetics.

## Materials and Methods

### Chemicals

We purchased 4-MBC (Eusolex 6300, CAS no. 36861-47-9, molecular weight 254.37, purity 99.7–99.9%) from Merck Schweiz (Dietikon, Switzerland) and 3-BC (Unisol-S22, CAS no. 15087–24–8, molecular weight 240.0, purity > 97.0 %) from Induchem AG (Volketswil, Switzerland).

### Animals

Long Evans rats were purchased from Centre d’Elevage R. Janvier (Le Genest-St. Isle, France) and shipped to the animal facility of the Department of Physiology, Medical School, Complutense University, Madrid, Spain. The animals were kept under controlled conditions with free access to water and a soy-free rat chow (Nutreco, Toledo, Spain). Animal experiments followed the European Union normative (86/609/EEC; European Union 1986) and were approved by the Institutional Animal Care Committee of Complutense University. All animals were treated humanely and with regard for alleviation of suffering.

### 4-MBC or 3-BC treatment

Chow was prepared by adding 4-MBC and 3-BC to the soy-free rat chow (Nutreco). 4-MBC was added at concentrations of 10 and 100 mg/kg chow, yielding an average daily intake of 0.7 and 7 mg/kg body weight (bw)/day, and 3-BC was added at concentrations of 1 and 3.3 mg/kg chow, yielding a dose of 0.07 and 0.24 mg/kg bw/day. Control chow consisted of the same matrix.

This study was part of a larger study involving dissection of brain and reproductive organs at different postnatal stages and was designed to mimic exposure through the food chain during prenatal and early postnatal life. Males and females of the parent generation (F_0_; 5–6 weeks of age) were fed for at least 10 weeks before mating (including one spermatogenic cycle) with chow containing 4-MBC (0.7 or 7 mg/kg bw/day) or 3-BC (0.07 or 0.24 mg/kg bw/day) or control chow. Treatment continued throughout pregnancy and lactation. Rats were allowed to mate overnight, and pregnant (sperm-positive) females were kept in groups of two and separated 1 day before parturition.

### Birth and tissue preparation

Around expected birth term [gestational day (GD) 23; GD1 = 24 hr after mating], we observed pregnant dams at least three times during the light period for signs of birth. The day of birth was defined as postnatal day (PND) 1 (GD23). The dam and litter remained undisturbed until all the offspring had been born. Then one or two of the male offspring were gently taken out of the cage, wrapped in warm paper towels, and kept under red light (32 ± 1°C) for 1–2 min before being anesthetized by cooling the body until motionlessness in crushed ice. After decapitating the rats on a precooled plate (ice), the lower part of the body was sectioned just below the level of the umbilical cord using a razor blade and immediately immersed into 4% buffered formaldehyde for 24 hr and stored in 70% ethanol. One offspring per litter was transferred to the University of South Dakota for final tissue preparation, three-dimensional (3-D) reconstruction, and morphometric analysis.

### 3-D reconstruction

The urogenital sinus (UGS) and developing prostate were processed as described previously (Timms et al. 2005). Briefly, serial tissue sections (6 μm) were stained with hematoxylin and eosin and photographed with an Olympus DP70 digital camera fitted to an Olympus BX60 microscope (Leeds Precision Instruments, Minneapolis, MN, USA). Histological images of the developing prostate buds and associated structures were traced, contoured, and realigned as surface-rendered 3-D models using Winsurf reconstruction software (University of Hawaii at Manoa, Honolulu, HI, USA). To compare and examine the effects of the treatments with untreated control animals, we performed morphometric and volumetric analyses.

### Statistics

All volumetric and ductal development data, with the exception of the mesenchymal study, were analyzed using one-way analysis of variance, followed by a Dunnett’s post hoc test, using StatMost for Windows software (DataMost Corporation, Salt Lake City, UT, USA). We analyzed the mesenchyme volume data using the Mann-Whitney test. The confidence level for rejecting the null hypothesis was *p* < 0.05. Data are presented as mean ± SE. For all groups, *n* = 4. All samples were blinded to ensure the absence of bias.

## Results

### Accessory sex glands

**Figure 1:**
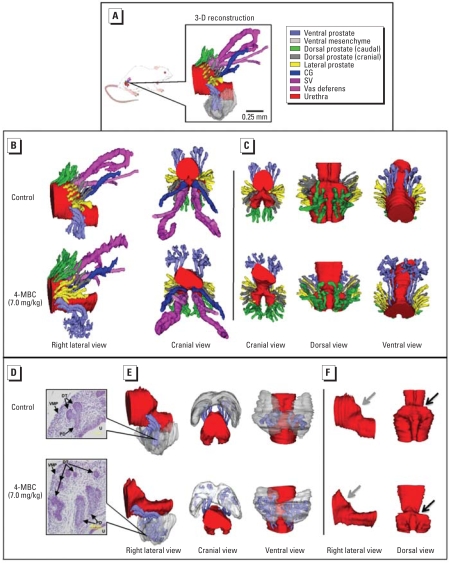
3-D serial section reconstruction of accessory reproductive organs in male PND1 Long Evans rats after systemic and developmental *in utero* exposure to 7.0 mg/kg bw 4-MBC. Abbreviations: DT, distal tip; PD, proximal duct, VMP, ventral mesenchymal pad. (*A*) Right lateral view of the surface-rendered anatomical reconstruction of the UGS and ASG structures in a control male. Individual structures are identified by color. (*B*) Reconstruction of the right lateral and cranial views of the UGS from a 4-MBC–treated male and untreated control male illustrating the significant regional growth differences in ducts of the caudal dorsal prostate and branching morphogenesis development in the ventral region. (*C*) Additional anatomical views of the prostatic ducts of a control male and a 4-MBC treated male showing the regional growth patterns. (*D*) Representative histological views of distal-tip budding in the ventral prostate region. Initial bifurcation is the primary feature of the control male, whereas extensive secondary branching morphogenesis has occurred in the 4-MBC–treated male (stained with hematoxylin and eosin; bar = 100 μm). (*E*) Anatomical view of the UGS, ventral ducts, and VMP showing that the distal tips of the ducts in the control male have made initial contact with the VMP. In the 4-MBC–treated male, the distal tips have penetrated the mesenchymal tissue and undergone later stages of branching morphogenesis. (*F*) Lateral and dorsal view of the UGS. Shape of the UGS (gray arrow) and bladder neck region of the urethra (black arrow) are changed in the 4-MBC–treated male.

**Figure 2:**
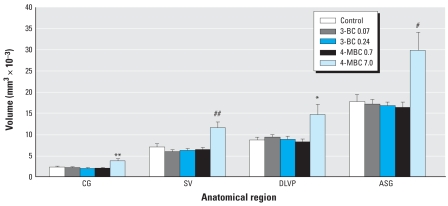
Comparative volume analyses of individual and combined regions of the ASGs in PND1 males after prenatal exposure to various doses of UV filter compounds. Significant effects were observed only in the 7.0 mg/kg bw–treated males. Values shown are mean ± SE (*n* = 4 for all groups). *p < 0.05, **p < 0.01, ^#^*p* < 0.005, and ^##^*p* < 0.001 compared with control males.

Figure 1A illustrates the components of the accessory sex glands (ASGs) that were examined individually or as anatomically related groups. The ASGs, including the seminal vesicles (SV), coagulating gland (CG), and the dorsal, lateral, and ventral prostate (DLVP) as a whole, were significantly sensitive to the effects of 4-MBC at the 7.0-mg/kg bw dose (Figure 2). All of the subsequent findings pertain to the effects of exposure to this dose of 4-MBC compared with untreated control males. No significant effects were observed under any 3-BC treatments or the 0.7-mg/kg bw dose of 4-MBC.

We observed a 69% overall increase in the ASG volume (*p* < 0.005), compared with untreated controls. Treatment significantly increased the volume of the SV by 66% (*p* < 0.001), the CG by 68% (*p* < 0.01), and the developing prostate (DLVP) by 69% (*p* < 0.01; Figure 2). We also observed a narrowing of the urethra at the neck of the bladder in this treatment group (Figure 1F). This feature is consistent with a prior study of endocrine disruptor exposure (Timms et al. 2005).

### Dorsolateral prostate

A detailed 3-D reconstruction of a specific group of ducts in the developing dorsolateral prostate revealed that the most sensitive region to 4-MBC was the caudal area of dorsal prostatic ducts, which increased in volume by 62% (*p* < 0.05; Figures 1A,C and 2). In contrast, the cranial portion of the dorsal prostate and the lateral prostate did not exhibit any significant growth response to treatment.

### Ventral prostate and ventral mesenchymal pad (VMP)

**Figure 3:**
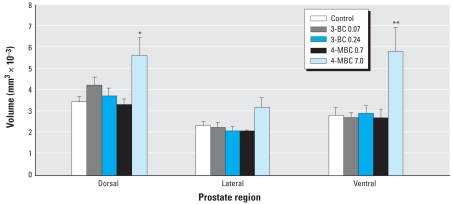
Comparative volume analyses of specific regions of the developing prostate in PND1 males after prenatal exposure to various doses of UV filter compounds. Significant effects were observed only in the dorsal and ventral regions of the 7.0 mg/kg bw–treated males. Values shown are mean ± SE (*n* = 4 for all groups). *p < 0.05, and **p < 0.01 compared with control males.

We observed a significant increase in prostatic duct volume (106%; *p* < 0.01) in the ventral region of the prostate (Figure 3). The 3-D reconstruction analysis also revealed a more pronounced development of distal tip branching morphogenesis (Figure 1B,C,D). The VMP (Timms et al. 1994), which is intimately involved in distal tip branching, was also increased in volume by 64% (*p* < 0.05; Figure 1F).

### Prostatic duct development

**Figure 4:**
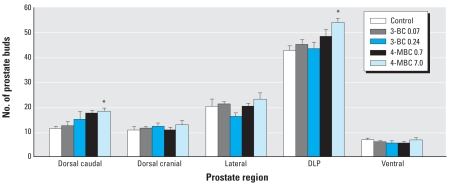
Effects of prenatal exposure to UV filter compounds on prostate duct development in PND1 males. A significant increase in the number of developing ducts was observed in the combined dorsolateral prostate region (DLP). This increase was due specifically to an increase in the number of ducts in the caudal region of the dorsal prostate. Values shown are mean ± SE (*n* = 4 for all groups). *p < 0.05 compared with control males.

The number of prostate ducts developing from the UGS has been shown to increase after prenatal estrogen treatment (Timms et al. 2005; vom Saal et al. 1997). In the present study we observed a similar increase in the number of dorsal ducts, particularly in the caudal region of the UGS (62%, *p* < 0.05; Figure 4). However, despite an increased ductal volume in the ventral prostate, there was no increase in the number of ducts growing from the ventral wall of the UGS (Figures 1C and 4).

## Discussion

Normal prostate development is dependent on androgens (Siiteri and Wilson 1974). However, exposure to abnormally high levels of estrogen during the critical period of development has the potential to alter growth-control mechanisms (Huang et al. 2004; Prins et al. 2006). In contrast, low-level exposure to estrogen, or estrogen mimics, during the same developmental period has been shown to alter the regulation of androgen receptors (AR) and ERs (Richter et al. 2007; vom Saal et al. 1997). A shift in receptor density predisposes the prostate for altered hormonal regulation (Wu et al. 2007). Along with changes in receptor regulation, endocrine-disrupting compounds inhibit the production of phosphodiesterase and increase transcription of the aromatase gene *CYP19* (cytochrome P450) under promotion of aromatase promoter II in the prostate (Fan et al. 2007). This aromatization of testosterone into localized free estrogen can further destabilize the androgen-regulated growth of prostatic epithelium.

The results of the present study support the classification of 4-MBC as an endocrine disruptor (Schlumpf et al. 2001, 2004b; Schreurs et al. 2005). The doses of 7 and 0.7 mg/kg bw/day examined in this study represent the LOAEL and NOAEL, respectively, for classical toxicity end points in extended one generation–type studies, such as puberty in males, adult ventral prostate weight, and female sexual behavior. Changes in estrogen-regulated gene expression in adult male and female offspring were still present at the lower dose, 0.7 mg/kg bw/day (Durrer et al. 2005, 2007; Maerkel et al. 2007; Schlumpf et al. 2008). In chronic (90-day) studies on adult ovariectomized rats, interactions of 4-MBC with estrogenic mechanisms have been observed in additional targets such as luteinizing hormone, bone, adipose tissue, and leptin. However, these effects were seen at higher doses than those used in our developmental toxicity studies (Seidlova-Wuttke et al. 2006a, 2006b).

Exposure of neonates to the chemicals has been assessed by determining 4-MBC and 3-BC concentrations in rat milk (Schlumpf et al. 2008). The effective dose of 7 mg/kg bw/day yields a 4-MBC concentration of 209 ng/g lipid, which is 11 times the highest value found thus far in our ongoing monitoring study on human milk (19 ng/g lipid). This level would be considered within the risk factor zone for human safety.

The prostate is very sensitive to deviations from homeostatic androgen-to-estrogen ratios. Compounds that mimic the effects of estrogen have the potential to alter the development of the prostate by inducing morphologic changes (Prins 1992; Timms et al. 2005; vom Saal et al. 1997). These effects follow an inverted U-curve pattern, which at low doses cause a proliferative response (Timms et al. 2005) and at higher doses cause an inhibitory effect (vom Saal et al. 1997). In the present study, using low-dose *in utero* systemic exposure, we observed proliferative effects similar to those found in other low-dose exposure experiments (Timms et al. 2005; vom Saal et al. 1997).

4-MBC is a preferential ER-β ligand with limited ER-α binding capacity *in vitro* (Mueller et al. 2003; Schlumpf et al. 2004a), but it also exerts estrogenic effects *in vivo* that depend on ER-α, such as the uterotrophic response in immature rats (Schlumpf et al. 2001, 2004a). This might be due to an estrogenic metabolite whose activity has not been characterized in detail (Völkel et al. 2006). Thus, *in vivo* effects of this UV filter probably represent a combination of ER-β– and ER-α–mediated actions. Reporter gene assays on AR yielded conflicting results for both 4-MBC and 3-BC, including complete absence of agonistic or antagonistic activity, in contrast to some other UV filters (Ma et al. 2003), as well as the presence of weak antiandrogenic activity (Schreurs et al. 2005). So far, interactions with androgenic mechanisms have not been tested in acute *in vivo* models, but estrogenic activity of 4-MBC and 3-BC has been confirmed in the uterotrophic assay (Schlumpf et al. 2001, 2004a; Tinwell et al. 2002). Thus, actions on ER seem to provide the most straightforward explanation for the effects of 4-MBC on the neonatal prostate. It is interesting that the dose of 7 mg/kg bw/day 4-MBC enhances prostate growth during the neonatal period but causes a decrease in the weight of ventral and dorsolateral prostate lobes and a reduced expression of AR and ER-α at mRNA and protein levels in chronically exposed adult offspring (Durrer et al. 2007; Schlumpf et al. 2008). Because the adult rats had been continuously exposed during prenatal and postnatal life, it seems possible that the different effects are due to a switch in the relative importance of the actions on ER-α and ER-β. The consequences of this switch, with regard to potential lifelong exposure, should be kept in mind (Durrer et al. 2007; Schlumpf et al. 2008).

The lack of effect of 3-BC was surprising because 3-BC and 4-MBC behave very similarly in acute tests for estrogenic activity (Schlumpf et al. 2001, 2004a). The 3-BC doses we used affect gene expression in the early post-natal uterus (Schlumpf et al. 2008), but they may be too low to influence prostate growth. However, in spite of similarities in chemical structure and acute actions, the effect patterns of 4-MBC and 3-BC differ in developmentally exposed adult animals (Durrer et al. 2007; Schlumpf et al. 2008). Ventral prostate weight was reduced by 4-MBC in a dose-dependent manner, but it was unaffected by 3-BC except for the lowest dose used in that study (0.24 mg/kg bw/day). Also, effects of 3-BC and 4-MBC on AR and ER-α in prostate of adult offspring were opposite at mRNA and protein levels (Durrer et al. 2007; Schlumpf et al. 2008). Thus, it seems conceivable that effects of two compounds on the neonatal prostate may differ, even though the reason for this difference remains unknown.

The different growth effects within specific regions of the prostate substantiate earlier studies. Previously, the combined dorsal and lateral regions were grouped as the dorsolateral prostate (Prins and Korach 2007; Timms et al. 2005). Using our 3-D reconstruction program, Winsurf, we were able to further differentiate the dorsolateral prostate into its dorsal and lateral components. In addition, the dorsal prostate was divided into two distinct regions separate from the lateral prostate (Figure 1A). The effects seen in these areas were unique. We observed no significant volumetric changes in the lateral prostate or in the cranial region of the dorsal prostate ducts (Timms et al. 1994), a region described as LP1 by Hayashi et al. (1991). In the caudal region of the dorsal prostate ducts there was a significant increase in volume, which was primarily due to an increase in the number of ducts developing from the UGS wall (Figure 1C). In the normal rodent prostate the number of ducts that develop from the wall of the UGS is fairly constant (Timms et al. 2005). Any additional ducts formed then become part of the adult glandular structure and have the potential to increase the prevalence of disorders later in life (Risbridger et al. 2005).

**Figure 5:**
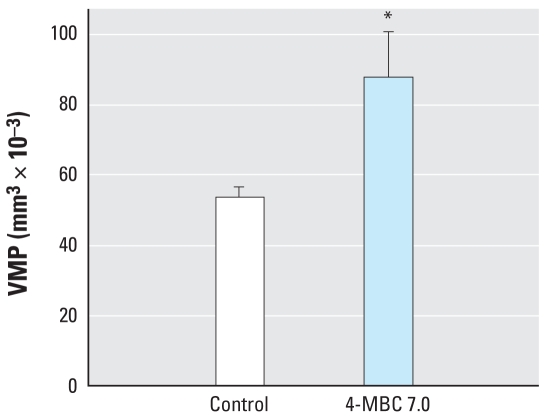
Effect of prenatal exposure to 4-MBC (7.0 mg/kg bw) on VMP tissue volume in PND1 males. Values shown are mean ± SE (*n* = 4 for all groups). *p < 0.05 compared with control males.

An important component of ductal formation is the surrounding mesenchymal tissue into which the prostatic buds grow (Cunha and Cooke 2004). Growth factors produced by this tissue play an important role in the process of branching morphogenesis and early glandular development (Thomson et al. 2002). Each region of the prostate and the adjacent Wolffian duct system contain their own respective mesenchyme (Hannema and Hughes 2007; Thomson et al. 2002; Timms et al. 1994, 1995, 1999). Branching morphogenesis is initiated after the buds grow into their local mesenchyme (Timms et al. 1995; vom Saal et al. 1997). Branching morphogenesis is the process by which the distal tips of immature prostatic ducts begin bifurcation and form the complex glandular architecture commonly seen in the adult prostate (Figure 1D). The increased ductal volume in the ventral prostate that resulted from an increase in the distal tip volume was a very different response to that seen in the dorsal ducts, where the increased ductal volume was primarily due to an increase in the number of ducts. These distinct regional differences suggest that exposure to endocrine disruptors during fetal development can affect specific developmental events, such as duct formation and branching morphogenesis. Furthermore, this suggests that environmental estrogens can exert their effects on distinct mechanisms within the same tissue. Because the VMP (Timms et al. 1994) is an important growth-inducing tissue in ventral prostate development (Thomson et al. 2002), we examined the volumetric parameter and found a significant increase in volume after 4-MBC exposure (Figures 1E and 5). The VMP is present before the onset of ductal outgrowth (Timms et al. 1994, 1995), and it is possible that the increased volume may have been established before ductal development in response to 4-MBC exposure. The increase in the number of caudal ducts in the dorsal prostate may reflect a growth effect of exposure via epithelial–mesenchymal interactions in the peri-urethral mesenchyme surrounding the urothelium. This mesenchyme is distinct from the VMP (Timms et al. 1995) and is likely to express different growth factors.

The recent evidence regarding the effects of 4-MBC on the developing fetus has placed this widely used UV filter in the category of known endocrine-disrupting compounds (Schlumpf et al. 2008; Søeborg et al. 2007). The effects of this compound on the developing prostate are strikingly similar to those of another known estrogen-mimicking compound, bisphenol A (BPA; Timms et al. 2005). UV filters, including 4-MBC, bio-accumulate in the environment (Balmer et al. 2005; Buser et al. 2006). They were found in 75% of human milk samples (Schlumpf et al. 2008), indicating potential transfer of these chemicals to the newborn infant. Human exposure stems mainly from the use of cosmetics, but exposure may also originate from the food chain. Both the European Union [Scientific Committee on Consumer Products (SCCP) 2006a, 2006b] and the U.S. Food and Drug Administration (2003) are currently reviewing data on 4-MBC, also known as enzacamene, Parsol 5000 (Roche 2000), and Eusolex 6300 (Merck 2005). The different responses observed in the present study suggest that the two developmental processes of ductal outgrowth and branching morphogenesis are affected independently by exposure to environmental chemicals at critical periods of UGS development. Our findings of prenatal growth effects, plus previous reports that these compounds are found in the environment, justify additional studies to determine the mechanisms underlying the characteristic growth responses during fetal development.
